# Unlocking the future of smart food packaging: biosensors, IoT, and nano materials

**DOI:** 10.1007/s10068-023-01486-9

**Published:** 2023-12-28

**Authors:** Aboli R. Bhatlawande, Pravin U. Ghatge, Gopal U. Shinde, R. K. Anushree, Sagar D. Patil

**Affiliations:** 1 Department of Food Chemistry & Nutrition, CFT, VNMKV, Parbhani, India; 2 PI-NAHEP-DFSRDA-ICAR, VNMKV, Parbhani, India; 3 Department of Food Science & Nutrition, CCS, VNMKV, Parbhani, India; 4 Department of Food Process Engineering, CAET, VNMKV, Parbhani, India

**Keywords:** Smart food packaging, Food safety, Improved food quality, Sustainable practices, Biosensors

## Abstract

This review examines how biosensors, the Internet of Things (IoT), and nano materials can revolutionize food packaging. It highlights the limitations of traditional packaging, particularly concerning barrier properties and food quality monitoring. The paper aims to provide specific insights into the potential of these technologies. Biosensors enable real-time monitoring and spoilage detection, ensuring safer products, while IoT enhances traceability and transparency in the supply chain, leading to reduced material waste, energy waste, and operational inefficiencies, ultimately improving efficiency. Nano materials offer improved barrier capabilities, strength, and antimicrobial properties, enhancing product quality and sustainability. The review paper also discusses the promising future of smart food packaging, driven by technological advancements and consumer demand for safer and eco-friendly products. However, it acknowledges the challenges related to regulations, sustainability, and consumer acceptance that need to be addressed for widespread adoption. In conclusion, this paper demonstrates how smart food packaging with biosensors, IoT, and nano materials can transform the food industry by overcoming traditional limitations and meeting evolving consumer needs, providing improved food safety, quality, and sustainability.

## Introduction

In today’s food industry, packaging serves as a linchpin, fulfilling multifaceted roles vital to safeguarding product quality and consumer safety. It acts as a shield, protecting against external elements like bacteria, insects, light, heat, oxygen, and odours, thus preserving food products during their shelf life. Beyond this core function, packaging takes on diverse shapes and sizes, optimizing logistical efficiency, while also serving as a means of communication with consumers. It conveys vital information through labels, branding, and designs, catering to the modern consumer’s desire for convenience and time-saving. For over half a century, plastic packaging has reigned supreme, owing to its versatility and cost-effectiveness, eclipsing traditional materials such as glass, metal, paper, and cardboard. However, this ubiquity of petroleum-based plastics has catalysed a cascade of environmental concerns due to their non-biodegradable nature and the pollution generated throughout their life cycle, from production to disposal. To redress these pressing issues, the industry is undergoing a seismic shift towards eco-friendly packaging alternatives, with biodegradable and bioplastic materials, derived from biomass and characterized by their biodegradability, gaining remarkable traction. The genesis of innovative packaging solutions is fuelled by a confluence of factors: population growth, evolving consumer preferences favouring safer and healthier food choices, conservation of the environment, and pioneering strides in fields like nanotechnology and biotechnology. This confluence has birthed inventive packaging paradigms, prominently among them, active and intelligent packaging. Active packaging actively engages with food products, creating communication channels with consumers. The corpus of literature dedicated to active packaging has surged significantly since 2015, while intelligent packaging has also been on a gradual ascent over this period. These packaging technologies share a unifying objective: optimizing the efficiency and security of the food supply chain, curtailing food loss, slashing waste, and mitigating the impact of unnecessary transit. It is noteworthy that various publications have spotlighted the laudable performance of active and intelligent food packaging solutions in recent years (Altaf et al., 2018).

**Fig. 1:**
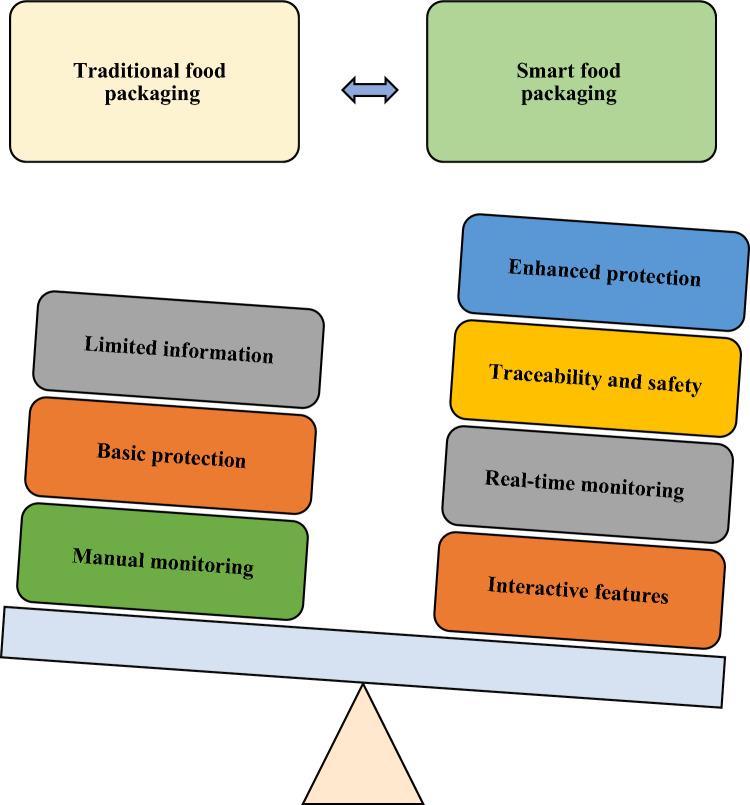
(Number line 60) Traditional food packaging Vs Smart food packaging

**Fig. 2:**
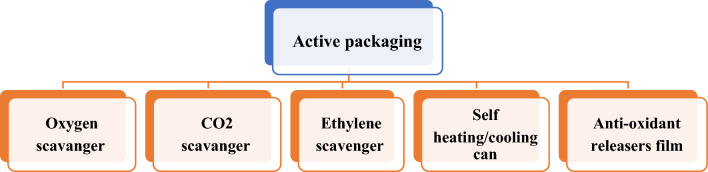
(Number line 89) Types of Active Packaging

**Fig. 3:**
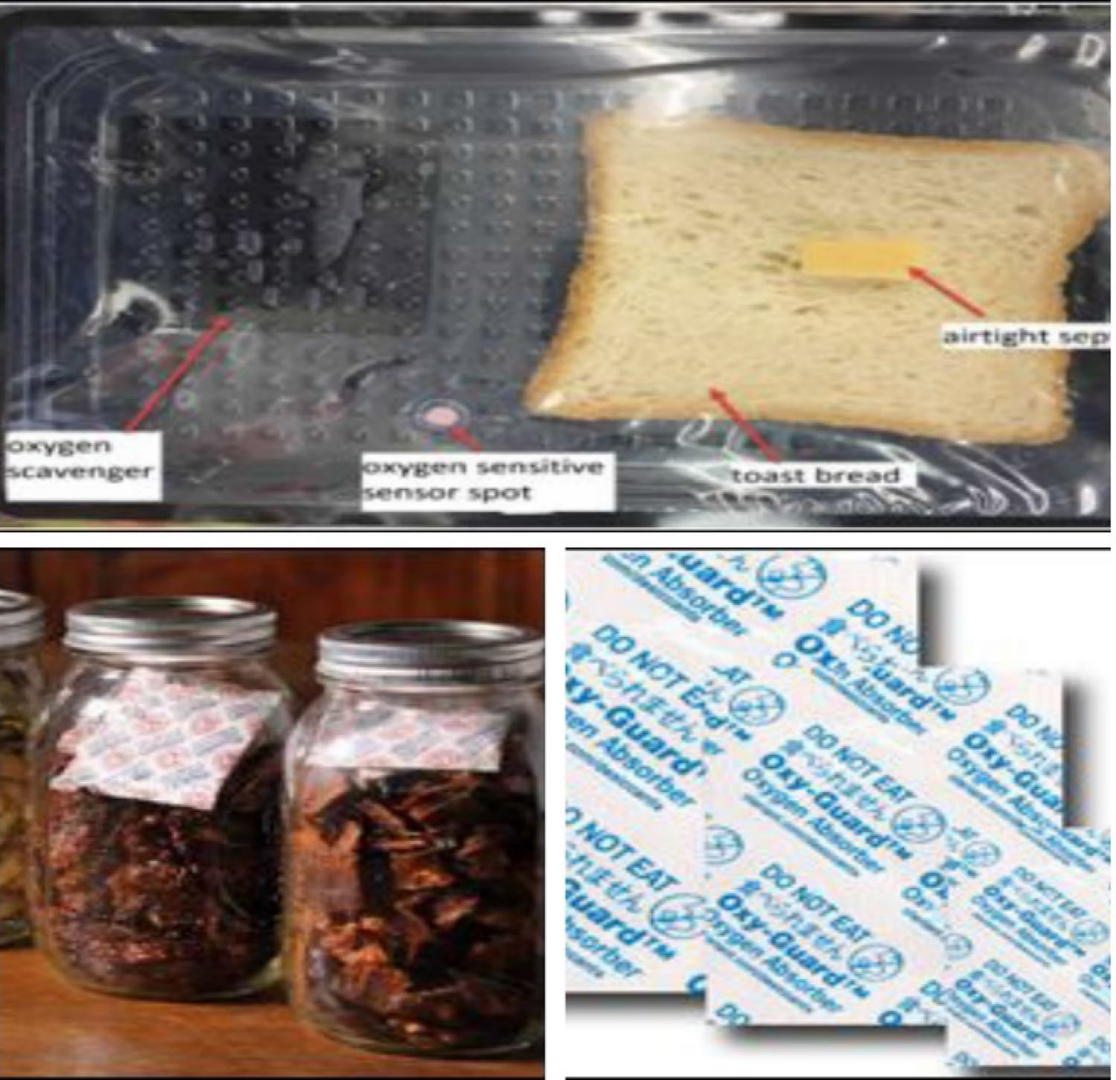
(Number line 113) Oxygen Scavenger Food Packaging (Ozcan, 2020; https://www.azom.com/news.aspx?newsID=57799)

**Fig. 4:**
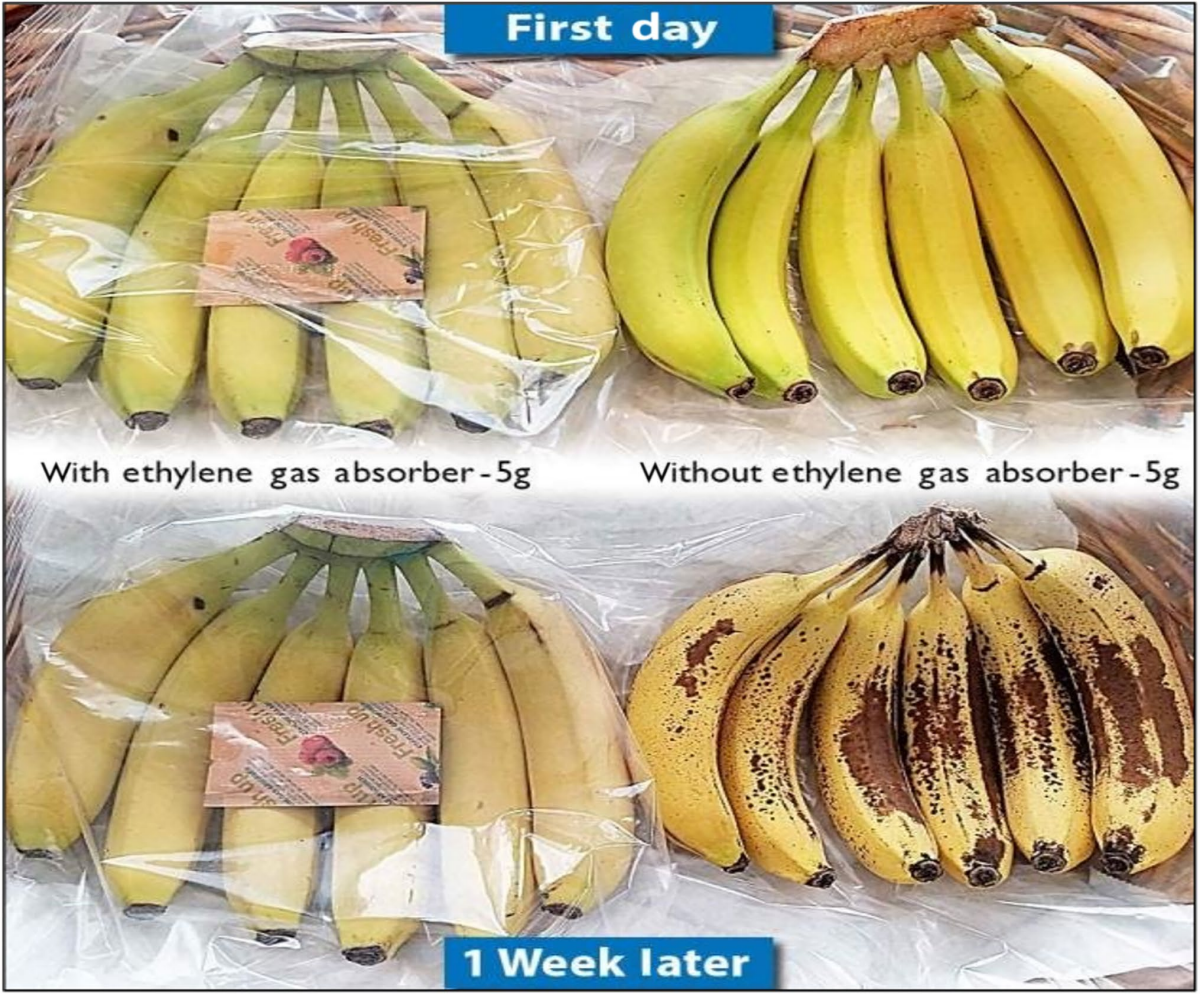
(Number line 142) Food packaging by using ethylene scavenger (Ozcan, 2020)

**Fig. 5:**
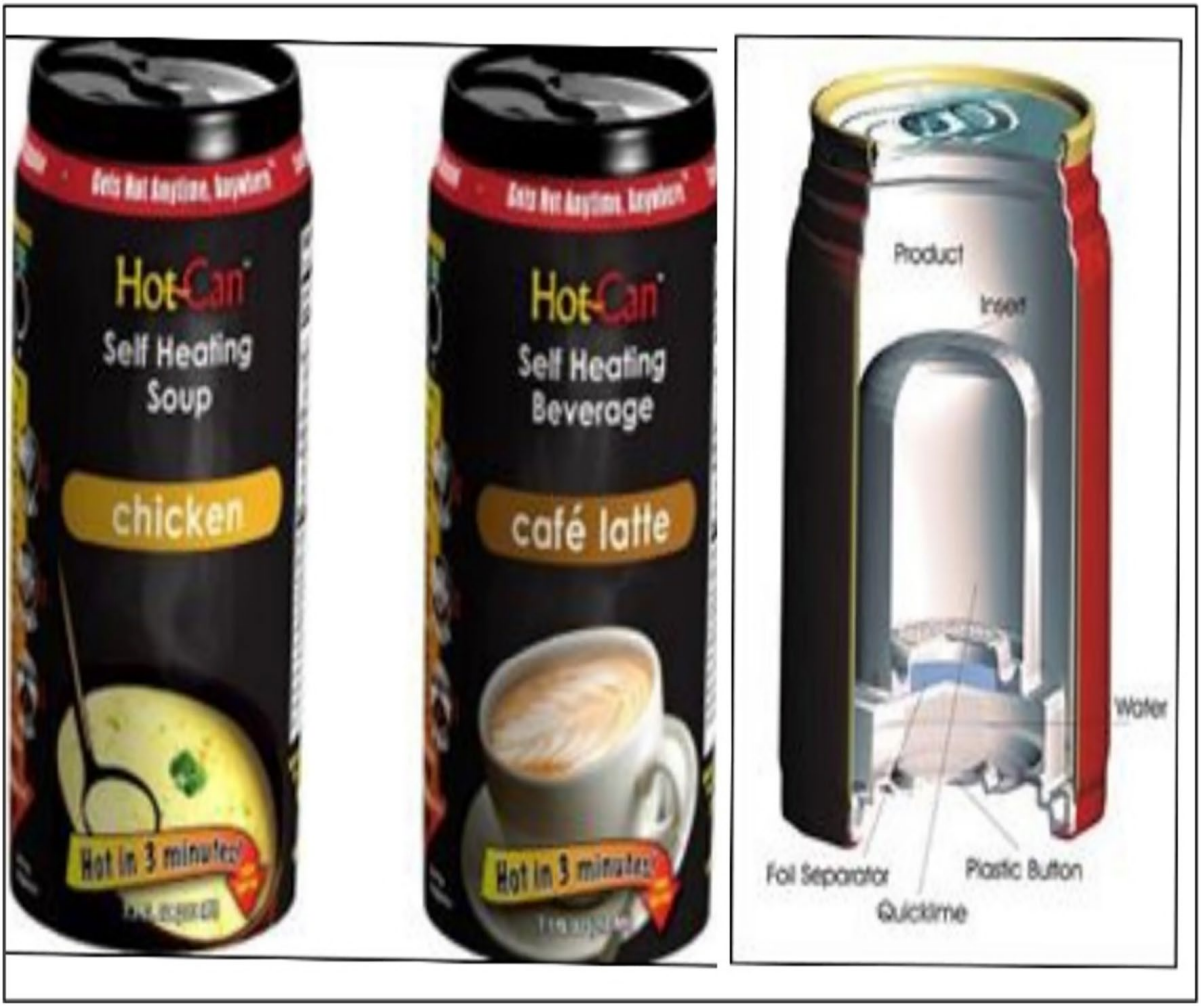
(Number line 163) Active packaging self heating can (https://newatlas.com/hot-can-self-heating-beverages/25646/)

**Fig. 6:**
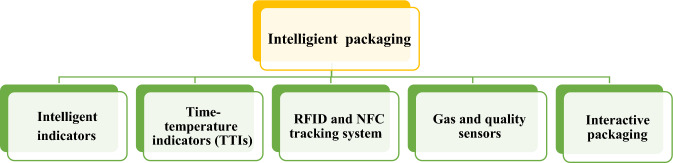
(Number line 200) classification of Intelligent Packaging

**Fig. 7:**
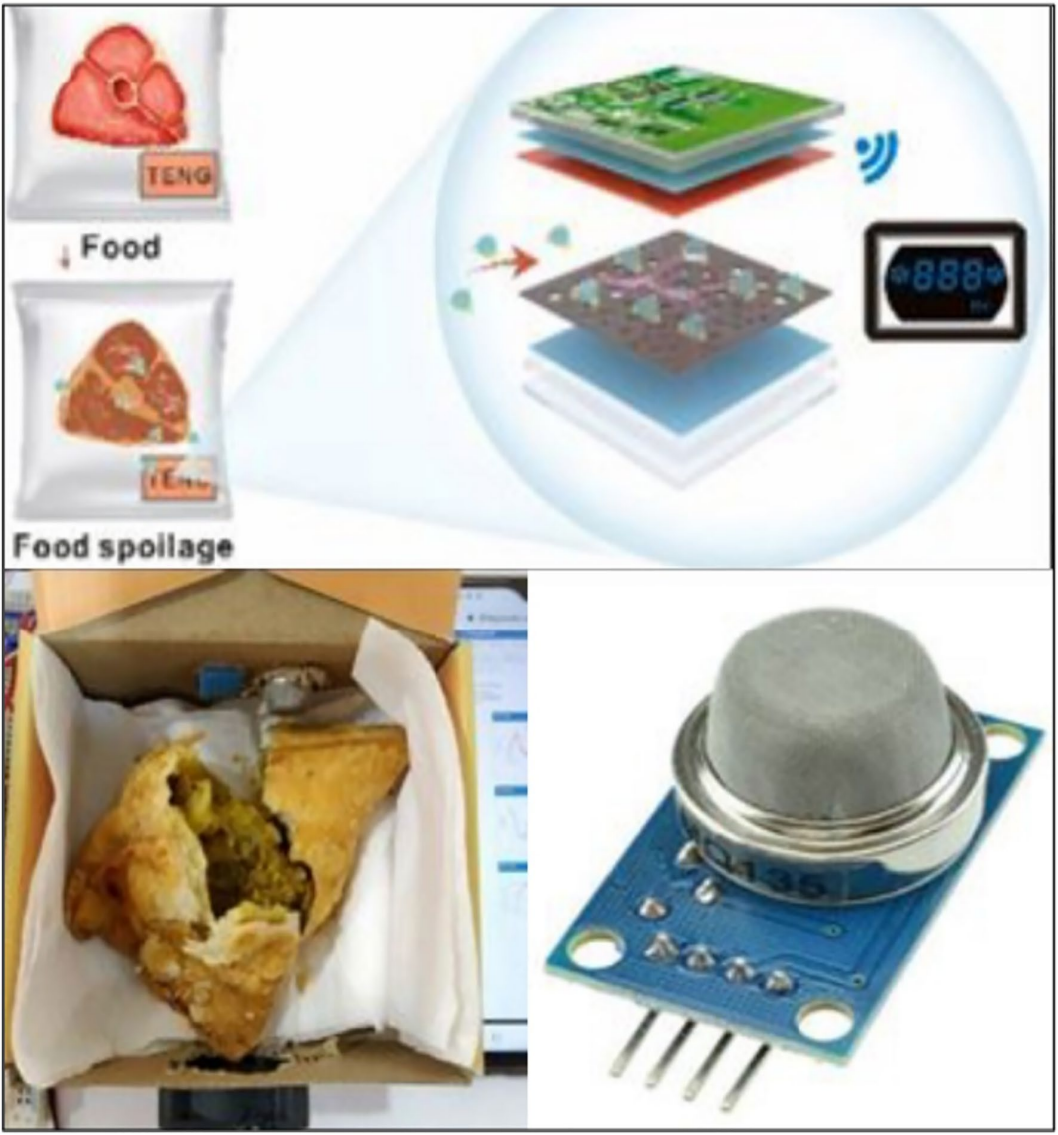
(Number line 256) IoT based gas sensor for quality assessment (Cai et al., 2021; https://iotdesignpro.com/projects/iot-based-food-monitoring-system; https://www.amazon.in/Robocraze-MQ-136-Gas-Sensor-Hydrogen/dp/B07F6YJJP5)

**Fig. 8:**
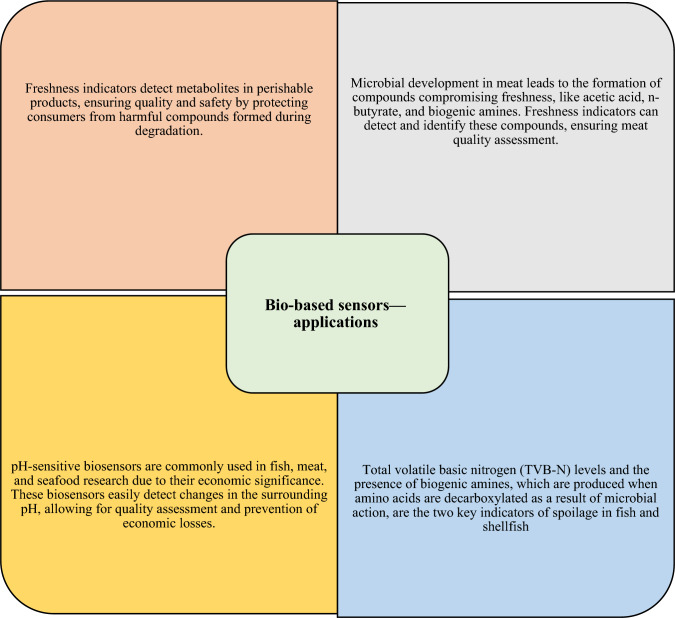
(Number line 296) Biobased sensor application

**Fig. 9:**
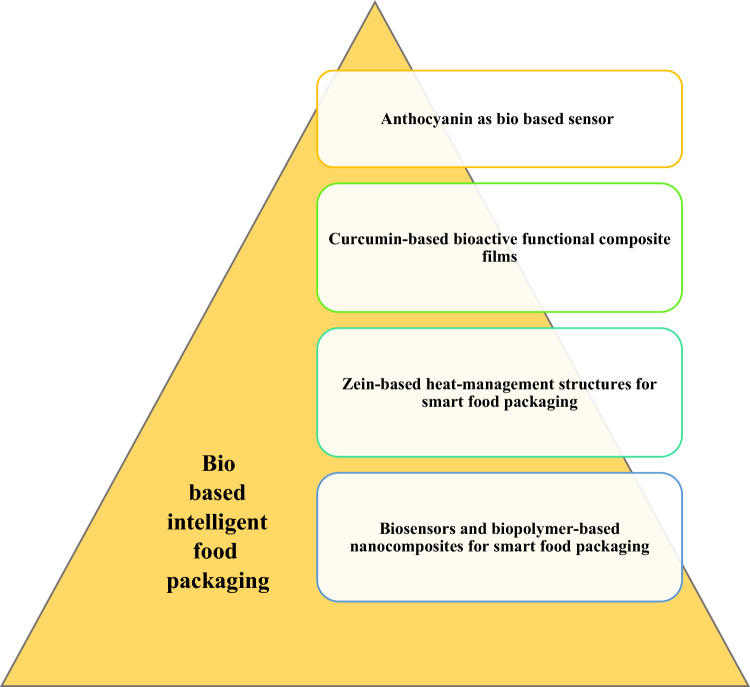
(Number line 320) Biobased Intelligent Food Packaging

**Fig. 10:**
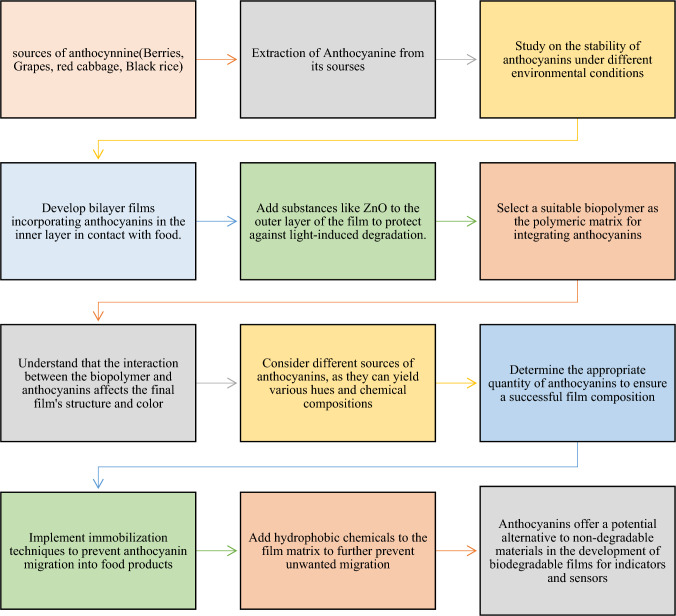
(Number line 348) Flowsheet of Anthocyanin as Bio based Sensor

**Fig. 11:**
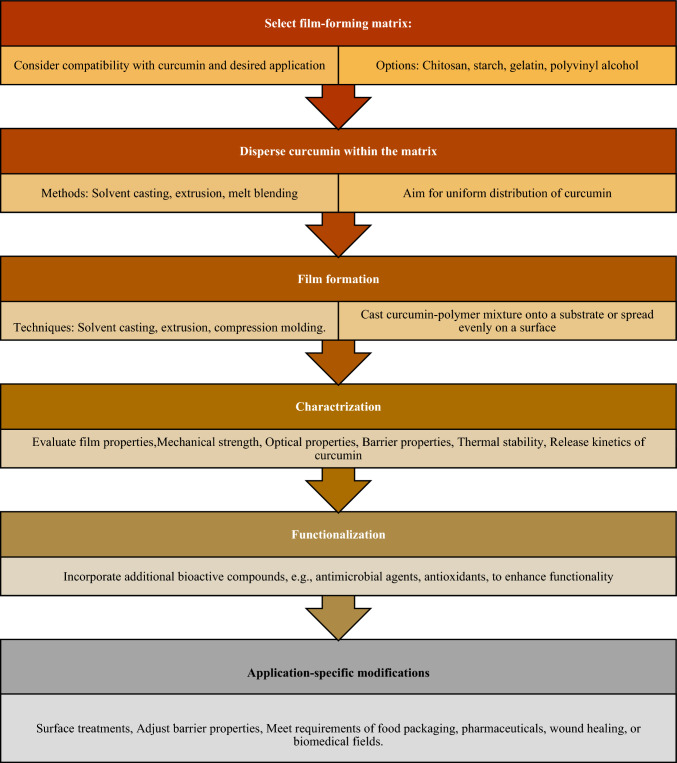
(Number line 367) Flowsheet of Curcumin-based bioactive functional composite film

**Fig. 12:**
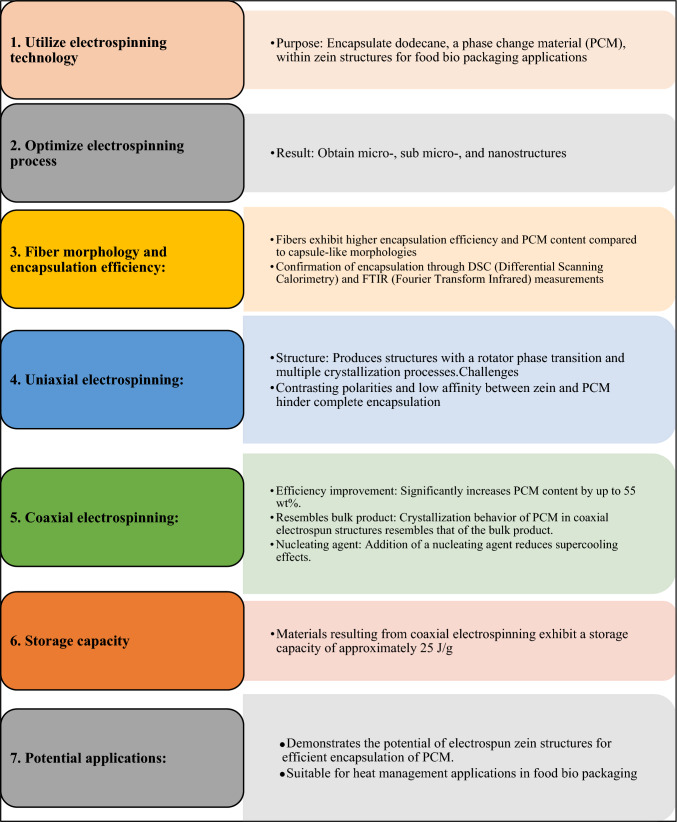
(Number line 384) Flowsheet of Zein-based heat-management structures for smart food packaging

To encapsulate, this review delves into these pioneering innovations, illustrating how biosensors, the Internet of Things (IoT), and nano materials are reshaping the landscape of smart food packaging. We explore their integration within the broader context of sustainable alternatives and the burgeoning realm of active and intelligent packaging. Ultimately, we shine a light on their transformative potential, propelling the food industry towards heightened supply chain efficiency, reduced waste, and the delivery of food products that are both safer and healthier, in alignment with the contemporary consumer’s expectations and environmental stewardship (Figs. 1, 2, 3, 4, 5, 6, 7, 8, 9, 10, 11, 12).

### Smart food packaging

Anything that offers “something extra” in addition to food containment and protection is considered smart food packaging. These “extras” can include anything from displays for monitoring pH, temperature, moisture, and freshness to a tracking device or extended shelf life. Although this system for food packaging is still in the early stages of development, it can track and trace products through blockchain technology and improve targeted recalls in addition to monitoring the freshness of meals and communicating that information to consumers. Smart packaging analyses storage conditions, food quality, and the inside/outside environment of the package using a variety of sensors, indicators, and smart levels.

### Market for smart packaging

According to a survey by the USDA, adults in the United States consumed more packaged goods in recent years compared to the past. The average monthly consumption of packaged goods increased by 26% between 2007–2008 and 2015–2016. The market for intelligent food packaging in the US is projected to reach $1.5 billion in 2019, while the global market demand for smart packaging was estimated to be $35.33 billion in 2018. The US market for smart packaging is expected to grow to approximately $3.6 billion in the coming decades, with Japan having the second-largest market worth $2.36 billion. Australia, the UK, and Germany are also expected to have significant demand for smart packaging. Food packaging refers to the use of advanced materials and technology to enhance the quality, safety, and shelf life of food products. It involves the monitoring and maintenance of the freshness, integrity, and overall quality of packaged food through sensors, indicators, monitoring systems, and other cutting-edge technologies (Sobhan et al., 2021).

#### Active packaging

Active packaging involves the incorporation of active substances or components into the packaging material, which interact with the food to extend its shelf life and maintain its quality. For example, oxygen scavengers can be included to remove oxygen from the package, thereby slowing down the oxidation process and preventing spoilage. Similarly, moisture absorbers or emitters can regulate humidity levels, preventing moisture-related issues such as mold growth or product drying (Altaf et al., 2018).

### Oxygen scavenger

Oxygen scavenging technologies aim to remove oxygen from the environment, particularly in food packaging. Different approaches are utilized, including iron powder oxidation, ascorbic acid oxidation, catechol oxidation, photosensitive dye oxidation, enzymatic oxidation, unsaturated fatty acids, and immobilized yeast on a solid material. The most common method currently employed is based on iron oxidation. However, incorporating oxygen scavenger sachets in food packaging poses some drawbacks, such as the risk of accidental ingestion by consumers and potential contamination if the sachet leaks. Additionally, the sachet requires a free flow of air to scavenge oxygen effectively. To address these issues, oxygen scavengers can be integrated into packaging materials like polymer films, labels, crown corks, and liners in closures. These oxygen scavenging materials offer the advantage of being suitable for all types of products, including liquid items (Altaf et al., 2018).

**Advantages**

- Extends the shelf life of oxygen-sensitive products like food and pharmaceuticals by removing or reducing oxygen levels inside the package.
- Prevents oxidation and spoilage, maintaining the freshness and quality of the contents.
- Enhances product safety by reducing the growth of aerobic bacteria.

**Disadvantages**

- May not be suitable for products that require oxygen, like certain fresh produce or oxygen-dependent bacteria.
- Costly compared to traditional packaging methods.

#### Practical example

Oxygen scavengers are commonly used in the packaging of snacks, dried fruits, and processed meats to maintain product freshness and reduce the risk of rancidity.

### CO_2_ scavenger

Carbon dioxide (CO_2_) is generated in certain foods as a result of deterioration and respiration reactions. It is essential to remove the accumulated CO_2_ from the food package to prevent food spoilage and potential damage to the packaging. For instance, fresh roasted coffee releases significant amounts of CO_2_ due to a reaction called Strecker degradation involving sugars and amines. If left unremoved, the CO_2_ can cause the packaging to burst due to increased internal pressure. Kimchi, a fermented vegetable product, is another food that produces CO_2_ during its fermentation process. Since kimchi cannot be pasteurized without compromising its sensory quality, the fermentation continues, leading to the production of CO_2_. The accumulation of CO_2_ within the packaging can cause it to balloon or even burst, necessitating the use of scavengers. The common method to scavenge CO_2_ involves using calcium hydroxide, which reacts with CO_2_ in the presence of sufficient water activity to form calcium carbonate (Altaf et al., 2018).

$${\text{Ca}}\left( {{\text{OH}}} \right)_{{2}} \, + \,{\text{CO}}_{{2}} \, \to \,{\text{CaCO}}_{{3}} \, + \,{\text{H}}_{{2}} {\text{O}}.$$

### Ethylene scavenger

To maintain the quality and extend the shelf life of fruits and vegetables, it is important to prevent the accumulation of ethylene gas in their packaging. Potassium permanganate-based ethylene adsorbers are commonly used for this purpose. The oxidation of ethylene with potassium permanganate occurs in two steps. Initially, ethylene is oxidized to acetaldehyde, which is further oxidized to acetic acid. Acetic acid can then be oxidized to carbon dioxide and water. Potassium permanganate adsorbers change color from purple to brown as the MnO_4_- is reduced to MnO_2_, indicating the remaining adsorbing capacity. The purpose of using these adsorbers is to prevent excessive ripening and softening of fruits and vegetables. They find applications in various produce, including apples, apricots, mangoes, tomatoes, avocados, carrots, potatoes, and Brussels sprouts.

Examples of ethylene adsorbers include sachets containing aluminium oxide and potassium permanganate, activated carbon with a metal catalyst, and clay-based materials. These adsorbers help control ethylene levels and maintain the quality of fruits and vegetables during storage (Altaf et al., 2018).

### Self heating/cooling can

A self-heating can is an advanced version of a regular food can that allows for heating the contents without external heat sources. It consists of two chambers: an inner chamber holding the food or drink and an outer chamber housing chemicals that undergo an exothermic reaction. When the user desires to heat the contents, they break a barrier that separates the chemicals in the outer chamber from the water or beverage in the inner chamber. There are two types of self-heating cans: one where the chemicals are in the outer chamber and water surrounds it, and another where the chemicals are in the inner chamber and the beverage surrounds it. The design offers benefits such as improved efficiency (reduced heat loss to the surrounding air) and prevents excessive heating of the product’s exterior, ensuring user comfort. These cans are typically made of aluminium or steel. The heating agent and reaction used vary depending on the product, but calcium oxide (CaO) is commonly employed. The reaction involves the combination of calcium oxide with water to form calcium hydroxide (Ca(OH)_2_). Alternatively, copper sulphate and powdered zinc can be used, but this process is less efficient, resulting in the formation of zinc sulphate (ZnSO_4_) and copper (Cu).

There are also self-cooling cans, which are also made of aluminium or steel. These cans utilize liquid nitrogen or other cooling gases. They incorporate a Heat Exchange Unit (HEU) that enables rapid chilling of the beverage. By pressing a button at the base of the can, the environmentally safe reclaimed CO_2_ or cooling gas contained in the HEU is activated, leading to the cooling of the beverage in under a minute (Altaf et al., 2018).

### Anti-oxidant releasers film

An antioxidant releaser film is a component of active packaging that releases antioxidants into packaged food to prevent or slow down oxidation. Active packaging goes beyond traditional functions and actively interacts with the product to enhance quality and extend shelf life. The film is integrated into the packaging material or placed in direct contact with the food. It contains antioxidant substances, either natural extracts or synthetic antioxidants, which are gradually released over time. This creates a protective environment around the food, inhibiting oxidation. Antioxidant releaser films are used to maintain food quality by preventing oxidative reactions that cause flavor changes, color degradation, nutrient loss, and overall product deterioration. The released antioxidants scavenge free radicals and disrupt the chain reactions responsible for oxidation. The composition and design of these films vary based on the food and desired application. Different materials, coatings, or layers are used to ensure controlled release and compatibility with the packaged food. Antioxidant releaser films are an important part of active packaging, effectively preserving the quality and extending the shelf life of various food products (Altaf et al., 2018).

**Advantages**

- Inhibits the growth of bacteria, fungi, and other microorganisms, reducing the risk of spoilage and foodborne illnesses.
- Extends the shelf life of perishable food products, reducing food waste.
- Enhances food safety and quality, especially in ready-to-eat products.

**Disadvantages**

- Potential for the development of antimicrobial resistance if used excessively.
- Regulatory challenges in some regions due to concerns about consumer safety.

#### Practical example

Antimicrobial agents like silver nanoparticles or organic acids are incorporated into food packaging, particularly in products like fresh meat and seafood, to inhibit microbial growth and maintain product safety.

Active packaging methods offer innovative solutions to extend the shelf life, enhance the quality, and improve the safety of various products. However, the choice of active packaging method depends on the specific needs and characteristics of the product. Careful consideration of the advantages and disadvantages is essential to ensure that active packaging effectively serves its intended purpose while minimizing potential drawbacks.

### Intelligent food packaging

Intelligent food packaging refers to the use of advanced technologies and materials in packaging systems to enhance the safety, quality, and shelf life of food products. It incorporates various features such as sensors, indicators, and tracking systems to monitor and communicate information about the condition of the packaged food.

#### Intelligent indicators

Sensors embedded in the packaging are a crucial component of intelligent food packaging. These sensors can detect and monitor various parameters such as temperature, humidity, gas composition, and pH levels inside the package. By continuously monitoring these factors, the packaging can provide real-time information about the food’s freshness and quality. For example, if the temperature inside the package exceeds a certain threshold, the sensor can trigger an alert indicating a potential loss of quality or spoilage. Smart food packaging often includes intelligent labels or indicators that provide real-time information about the condition of the packaged food. These labels can have indicators that change color in response to specific factors such as temperature, time, or gas composition, indicating the freshness or spoilage of the food. For instance, a color-changing label can alert consumers if the food has been exposed to high temperatures, indicating a potential loss of quality or safety Thermochromic inks are used to indicate the ideal drinking temperature for a product by changing color in response to temperature changes. These inks can be printed on labels or containers that are heated or cooled before consumption. The color change activation range for thermochromic inks is typically from − 10 to 70 °C, and the ink reversibly changes color as it warms or cools. By selecting appropriate colors, hidden messages such as “DRINK NOW” or “TOO HOT” can become visible, providing a visual cue to the consumer (Altaf et al., 2018).

#### Time–temperature indicators (TTIs)

TTIs are a type of intelligent indicator that monitor the temperature history of the product during storage and transportation. They provide a visual or electronic indication if the food has been exposed to unfavourable temperature conditions, indicating a potential risk of microbial growth or loss of quality. This helps consumers and suppliers make informed decisions regarding the safety and freshness of the food (Altaf et al., 2018).

**Advantages**

- Provides real-time information about temperature fluctuations during transportation and storage, helping to maintain product quality.
- Alerts consumers and supply chain stakeholders if the temperature-sensitive product has been exposed to unfavourable conditions.
- Reduces the risk of selling or consuming spoiled products.

**Disadvantages**

- Limited to monitoring temperature-related issues and may not address other factors affecting product quality.
- Requires additional components and may increase packaging costs.

#### Practical example

Time–temperature indicators are commonly used in the transportation of vaccines, pharmaceuticals, and perishable food items like seafood to ensure that temperature-sensitive products are kept within safe temperature ranges.

#### RFID and NFC tracking systems

Radio Frequency Identification (RFID) and Near Field Communication (NFC) technologies are commonly used in smart food packaging to enable tracking and tracing of food products throughout the supply chain. RFID tags or NFC-enabled labels can be attached to the packages, allowing real-time monitoring of the product’s location, temperature, and other relevant data (Altaf et al., 2018). This improves traceability, reduces the risk of counterfeiting, and enhances food safety by facilitating quick recalls in case of contamination or other hazards.

#### Gas and quality sensors

Advanced packaging technologies can integrate gas and quality sensors that detect and measure parameters such as oxygen levels, carbon dioxide levels, humidity, pH, and volatile compounds within the package. This information helps determine the freshness, ripeness, and quality of the food. For example, gas sensors can identify the presence of ethylene gas, which is emitted by certain fruits and accelerates their ripening process. By detecting and removing ethylene, smart packaging can extend the shelf life of fresh produce (Sobhan et al., 2021).

**Advantages**

- Continuously monitor the freshness of perishable products, such as fruits, vegetables, and dairy items.
- Provide consumers with accurate information about the remaining shelf life or freshness status.
- Reduce food waste by encouraging consumers to consume products before they spoil.

**Disadvantages**

- Higher cost compared to traditional packaging methods.
- May require batteries or electronic components, increasing complexity.

#### Practical example

Freshness sensors are being developed for food packaging, where they can detect gases released by food spoilage and communicate this information to consumers through indicators or smartphone apps, helping consumers make informed decisions about product consumption.

#### Interactive packaging

Some smart food packaging incorporates interactive elements to engage consumers and enhance their experience. For example, packaging designs may include augmented reality (AR) features that provide additional information about the product, recipes, or nutritional facts when scanned with a smartphone. QR codes or quick response codes are also commonly used to provide access to product information, promotions, or loyalty programs. Intelligent packaging methods offer valuable insights and information to both consumers and supply chain stakeholders. They enhance the user experience, improve product safety, and reduce waste. However, the choice of intelligent packaging method should align with the specific needs and characteristics of the product, and the advantages and disadvantages should be carefully considered to ensure effective implementation.

### Developments in bio-based sensors

Research in the field of intelligent packaging focuses on bio-based sensors, which use natural substances and biopolymers as sensors in food packaging. The main focus is on freshness indications, which inform consumers about changes in food products or the environment, providing precise information about the food’s condition. Freshness indicators in intelligent packaging rely on visible color changes triggered by chemical or microbiological changes, usually associated with pH changes, signalling the decay of food quality. These freshness indicators can be direct or indirect. Direct indicators interact directly with specific metabolites or toxins, while indirect indicators react to changes in the food’s environment caused by chemical or microbiological changes. Synthetic dyes, although effective, are not suitable for food applications due to their toxic and harmful properties. Instead, natural colors derived from plants and fruits are preferred for their biodegradability, low toxicity, and minimal environmental impact.

When using natural color pigments as pH indicators, it is important to consider their stability and responsiveness to pH variations. Biopolymeric matrices incorporating natural color pigments have gained popularity in food packaging solutions, aligning with the trend of sustainable consumer choices. Certain natural pigments, such as methyl red, bromocresol purple, bromophenol blue, and chlorophenol red, have been proven effective in this context. Additionally, these natural pigments and biopolymeric matrices often possess bioactive qualities, making them useful not only as pH indicators but also as active ingredients in food products (Rodrigues et al., 2021).

### Bio-based sensors—applications

Food degradation due to microbial growth can lead to the production of metabolites that affect food freshness. Freshness indicators can detect the presence or synthesis of metabolites like volatile nitrogen molecules, carbon dioxide, biogenic amines, ethanol, or sulphurous compounds. Ensuring high-quality standards for perishable products such as fruits, vegetables, fish, and meat is crucial to prevent the formation of harmful metabolites during product degradation. For example, the freshness indicator can help identify compounds like acetic acid, n-butyrate, and biogenic amines that compromise meat quality during microbial development. In fish and shellfish, spoilage can be indicated by the levels of total volatile basic nitrogen (TVB-N) and the presence of biogenic amines, which are produced through microbial decarboxylation of amino acids. Metabolites such as ethanol or carbon dioxide (CO_2_) generated during food degradation also contribute to the deterioration of food quality, and freshness sensors can detect their presence. The use of biobased sensors utilizing natural substances in intelligent food packaging has been the focus of numerous studies, particularly in the context of fish, meat, and seafood, where quality degradation results in significant economic losses.

These pH-sensitive biosensors enable the detection of changes in the surrounding pH. Some compounds used in these sensors not only exhibit pH-sensitive color changes but also possess bioactive properties, such as antibacterial characteristics, which can enhance the preservation ability of the packaging material. However, incorporating these substances into the biopolymer matrix can impact the mechanical, barrier, and optical properties of the films. Therefore, a balance must be struck between the beneficial pH-sensing activity and the potential negative effects on the mechanical qualities of the biopolymer, depending on the specific application (Salgado et al., 2021).

### Anthocyanin as bio based sensor

Anthocyanins, natural pigments with antioxidant, antibacterial, and pH-sensitive properties, hold potential for developing bio-based sensors in smart packaging systems. However, ensuring their stability is crucial for their effective use as indicators. Anthocyanins are highly sensitive to temperature changes, with low temperatures being preferable to maintain their stability and prevent browning caused by high temperatures. They are also susceptible to degradation by oxygen and light. To overcome this limitation, bilayer films can be created with anthocyanins in the inner layer in contact with food and incorporating substances like ZnO in the outer layer to combat degradation from light exposure. The choice of a suitable biopolymer as the polymeric matrix is important for the integration of natural colors and sensor capabilities. The interaction between the biopolymer and the sensing substance influences the film’s structure and the anthocyanins’ color. Neutral biopolymers like starch and tara gum, which have minimal impact on the pigments’ color, are preferred. The source of anthocyanins also affects the color change in response to modifications in the food product, as different sources yield different hues and chemical compositions. The quantity of anthocyanins included in the film is also crucial for its success, as excessive aggregation can alter the matrix composition. To maintain the sensor activity of the pigments and prevent migration into food products, immobilizing them within the polymer matrix is recommended. Adding hydrophobic chemicals to the film matrix can prevent unwanted migration. Although immobilization restricts their antioxidant and antibacterial functions, anthocyanins can still scavenge, absorb, or interact with the portion of food in contact with the polymer. The inclusion of anthocyanins can also affect the mechanical and barrier properties of the films, and a well-balanced combination of reinforcements, plasticizers, and pigments can enhance the effectiveness of food packaging. Anthocyanins offer a potential alternative to non-degradable materials in the development of biodegradable films for indicators and sensors. Their stability, interaction with the polymeric matrix, and careful control of their quantity and immobilization are critical factors for successful implementation (Rodrigues et al., 2021).

### Curcumin-based bioactive functional composite films

Several studies have explored the incorporation of curcumin, a compound found in turmeric, into active and intelligent packaging films with promising results. Biopolymers, known for their biodegradability and functional properties, have gained significant interest in this area. Films combining curcumin with various biodegradable and biocompatible polymers have been developed, resulting in enhanced mechanical and barrier properties. The addition of curcumin has also demonstrated outstanding antioxidant and antibacterial activities against a range of common foodborne pathogens such as Escherichia coli, Salmonella spp., and Listeria monocytogenes in films made from pectin/chitosan blends and chitosan-based materials. Moreover, curcumin-loaded films have shown sustained and pH-dependent release of curcumin, making them suitable for controlled delivery applications. Researchers have observed improvements in mechanical, barrier, hydrophobic, and thermal stability properties of films without compromising transparency by incorporating curcumin. The intelligent packaging aspect of curcumin-based films allows for real-time evaluation of product quality through pH-indicating color changes. These films have proven effective in inhibiting lipid oxidation, limiting microbial growth, and ensuring food safety and quality. Overall, curcumin-infused active and smart packaging films hold promise for extending the shelf life of food products and enhancing their safety and quality (Roy et al., 2022).

### Zein-based heat-management structures for smart food packaging

Electrospinning technology was utilized to encapsulate dodecane, a phase change material (PCM), within zein structures for potential applications in food bio packaging. The optimization of the electrospinning process resulted in micro-, sub micro-, and nanostructures. The fibers exhibited higher encapsulation efficiency and PCM content compared to capsule-like morphologies. Encapsulation was confirmed through DSC and FTIR measurements. Uniaxial electrospinning produced structures with a rotator phase transition and multiple crystallization processes, while the coaxial approach significantly improved efficiency, increasing PCM content by up to 55 wt%. The crystallization behaviour of the PCM in coaxial electro spun structures resembled that of the bulk product. Furthermore, the addition of a nucleating agent reduced supercooling effects. Uniaxial electrospinning proved challenging for complete encapsulation due to the contrasting polarities and low affinity between zein and the PCM. However, coaxial electrospinning achieved higher efficiency and resulted in materials with a storage capacity of approximately 25 J/g. These findings demonstrate the potential of electro spun zein structures for efficient encapsulation of PCM and their suitability for heat management applications in food bio packaging (Pérez-Masiá et al., 2013).

### Biosensors and biopolymer-based nanocomposites for smart food packaging

Smart food packaging, a subject of increasing interest in the scientific community, leverages bio-nanocomposites, which offer several advantages like improved mechanical strength, better thermal properties, and enhanced gas barrier abilities. The incorporation of antimicrobial nanoparticles within these composites also helps inhibit the growth of microorganisms while further enhancing the packaging’s mechanical, thermal, and electrical properties. However, for the development of commercially viable smart food packaging, several factors must be considered. These include product cost, the ability of bio-nanocomposites to interact safely with food, and their capacity to prevent health-related issues. Biosensors, a crucial component of intelligent food packaging systems, have shown great promise, but their integration into packaging materials presents some challenges. Key considerations for biosensors include their microstructure, sensitivity, specificity, and detection limits. Despite manufacturers’ interest in biosensors for ensuring product quality, the commercialization of this technology is still in its early stages. To advance global smart food packaging technology, we need to address the issues associated with biosensors in packaging. Recommendations for biosensor development include achieving a small size and seamless integration with packaging materials, ensuring consistent sensitivity over time, and controlling costs for consumers. Safety concerns regarding biosensors and the particle sizes of bio-nanocomposites used in packaging should also be thoroughly evaluated. The use of biosensors and bio-nanocomposites in smart food packaging holds tremendous potential, but challenges and prospects require further exploration. A comprehensive understanding of biosensors and bio-nanocomposites is vital for creating sustainable and affordable smart packaging materials. This review offers an overview of current research and development in biosensors and bio-nanocomposites, emphasizing their benefits and limitations in the field of smart food packaging (Sobhan et al., 2021).

### Nano materials for smart food packaging

The development of intelligent packaging systems, which can provide real-time information about the quality and safety of food products, has gained significant attention in recent years. One promising approach involves the integration of biosensors and bio nano composites into food packaging materials. Biosensors, with their quick and reliable detection capabilities, offer the potential for monitoring the microbiological breakdown products of packaged food in real-time. Bio nano composites, on the other hand, exhibit excellent mechanical, thermal, optical, and antibacterial properties, making them suitable for creating smart packaging materials. However, several challenges and considerations need to be addressed for the successful implementation of biosensors and bio nano composites in smart food packaging. The size and structure of biosensors should be carefully designed to integrate with packaging materials and ensure long-term sensitivity and reliability. The overall cost of smart packaging should be kept under control to make it commercially viable. Safety aspects and ethical considerations related to biosensors’ potential impact on human health should be thoroughly evaluated. Bio nano composites, which combine biopolymers and nanoparticles, have shown promise in enhancing the functionality and performance of packaging materials. The particle sizes of bio nano composites must be proven safe for consumption before their incorporation into smart food packaging. Understanding the benefits and limitations of biosensors and bio nano composites is essential for developing sustainable and cost-effective smart packaging solutions.

The integration of biosensors and bio nano composites in smart food packaging holds great potential for ensuring food quality and safety. However, further research and development are needed to address challenges related to biosensor design, cost-effectiveness, safety, and ethical considerations. By advancing our understanding of these technologies, we can create intelligent packaging materials that contribute to the development of a smarter and more sustainable food packaging industry (Motelica et al., 2020).

### Safety concern

The use of smart packaging in the food industry requires strict regulations regarding materials, testing, and labelling. These regulations ensure that packaging components do not contaminate food or pose a risk to human health. It is important to clearly label components that should not be consumed, such as O_2_ removal sachets. Efforts are being made to eliminate the need for sachets by directly incorporating active ingredients into the packaging. Environmental regulations also need to be considered, addressing the usage, reuse, recycling, and identification of packaging materials to promote sustainability. Additionally, the potential risks of antimicrobial compounds used in smart packaging technologies, such as antimicrobial resistance, should be assessed to safeguard the food supply. Smart packaging offers numerous benefits to the food and beverage industries, including meeting consumer demands for convenient and longer-lasting products. Synthetic plastic materials have been widely used due to their affordability and functionality, but there is a growing focus on developing bioplastics to align with sustainability and environmental protection goals. Modifications in biopolymers through various treatments and additives have expanded their functionality for intelligent food packaging. However, many of these developments remain in the laboratory stage and need to be scaled up for commercial application. It is crucial to address interactions, regulations, safety concerns, and proper design to build consumer confidence in this technology. Future research should concentrate on evaluating the safety and performance of the entire packaging system when in contact with food. Natural bioactive substances are preferred, and research is underway to develop sensitive and specific quality indicators. The incorporation and controlled release of bioactive substances in biopolymer-based food packaging materials are being explored, and emerging technologies like nanotechnology packaging and antimicrobial packaging are being utilized to improve functionality. Regulations are essential for ensuring the safety and quality of smart food packaging. The development of bioplastics and their functional modifications hold promise for intelligent food packaging, but further research, scaling, and evaluation are needed. Advances in interactions, regulations, safety, and design will increase consumer confidence. Research is also focused on incorporating bioactive substances and improving packaging functionalities using emerging technologies.

In conclusion, smart food packaging has brought significant advancements to the food industry, enhancing food safety, quality, and sustainability. Real-time monitoring, sensors, indicators, and tracking systems have reduced food waste and improved the consumer experience. Future developments in sensors and data analytics will provide more accurate information about food quality and nutritional content. Integration with IoT platforms will improve supply chain management and traceability. Sustainability is a key focus, with research focusing on eco-friendly packaging materials and minimizing food losses. Smart packaging extends shelf life and ensures optimal conditions during storage and transportation. Embracing these innovations will lead to a more productive, sustainable, and consumer-centric food industry.
